# Insights from the ground: A qualitative investigation of retailer perspectives of the challenges and opportunities in the legal cannabis market in Newfoundland and Labrador, Canada

**DOI:** 10.1371/journal.pone.0333706

**Published:** 2025-10-22

**Authors:** Tanisha Wright-Brown, Dina Gaid, Maisam Najafizada, Elizabeth Schwartz, Thomas Cooper, William Newell, Lisa Bishop, Jennifer Donnan

**Affiliations:** 1 School of Pharmacy, Memorial University of Newfoundland, St. John’s, Newfoundland and Labrador, Canada; 2 Estimate Lab, St. Michael’s Hospital, Unity Health Toronto, Toronto, Ontario, Canada; 3 Faculty of Medicine, Memorial University of Newfoundland, St. John’s, Newfoundland and Labrador, Canada; 4 Department of Political Science, Memorial University of Newfoundland, St. John’s, Newfoundland and Labrador, Canada; 5 Faculty of Business Administration, Memorial University of Newfoundland, St. John’s, Newfoundland and Labrador, Canada; 6 Faculty of Business Administration, Memorial University of Newfoundland, Corner Brook, Newfoundland and Labrador, Canada; University of Durham: Durham University, UNITED KINGDOM OF GREAT BRITAIN AND NORTHERN IRELAND

## Abstract

**Background:**

The legalization of recreational cannabis in Canada has resulted in varying regulatory and market environments across provinces and territories. These differences shape how retail markets develop and how retailers perceive their opportunities, challenges, and roles in advancing public health objectives. In Newfoundland and Labrador (NL), cannabis retail operates within a distinctive framework shaped by centralized distribution, licensing requirements, and pricing regulations. This qualitative study explores how licensed and prospective retailers perceived the factors influencing the cannabis retail market in NL.

**Methods:**

Semi-structured virtual interviews were conducted with nine licensed and nine prospective cannabis retailers in NL. A thematic analysis, using Wright-Brown et al.’s Comprehensive Cannabis Retail Framework and Ritchie and Spencer’s framework analysis, was conducted. Both deductive and inductive coding were applied to identify framework-aligned and emergent themes.

**Results:**

Licensed retailers reported challenges such as restrictive advertising rules, high taxation, and supply chain inefficiencies, which they viewed as constraints on profitability and growth. At the same time, access to quality products, positive customer relationships, and informal mentorship networks were seen as enablers of success. Prospective retailers identified high licensing fees, limited access to opportunities, and financing difficulties as significant barriers to entering the legal market.

**Conclusion:**

This study highlights how NL’s cannabis retail system, designed to balance public health protection with market development, may inadvertently limit participation and business sustainability. The study illustrates how regulatory design can shape retailer experiences and market dynamics, underscoring the need to assess whether current regulations are achieving their intended outcomes. While focused on NL, these findings offer valuable insights for other jurisdictions with similar regulatory models, emphasizing the importance of aligning policy design with retailers’ experiences to foster a more inclusive, sustainable, and public health–oriented cannabis retail sector.

## Introduction

The global trend toward cannabis legalization and decriminalization has intensified debates over regulatory frameworks and their impact on public health, market viability, and consumer behavior. These frameworks are generally designed to reduce harm, control consumption, and curtail unlicensed markets [1–3]. However, despite growing scholarly and policy interest, limited attention has been paid to how these regulations affect the experiences and sustainability of key stakeholders, particularly licensed and prospective retailers. These perspectives are especially underrepresented in the literature, with marginalized communities often excluded from policy discourse [4,5].

This gap is particularly pronounced in smaller jurisdictions such as Newfoundland and Labrador (NL), where geographic isolation, economic constraints, and localized regulatory approaches shape the cannabis retail landscape in unique ways. Retailers in NL face distinct challenges in navigating the province’s regulatory environment, and their experiences offer critical insights into the development for more responsive and equitable cannabis policies. The exclusion of these voices from policy development risks unintended consequences, including restricted market access, threats to business viability, and potential compromises to public health and safety. A nuanced understanding of how regulatory and contextual factors influence these stakeholders is essential for evaluating the sustainability of legal cannabis businesses.

This study addresses this gap by examining how government regulations and broader environmental factors shape the experiences of cannabis retailers in NL. Guided by the central research question, “How do government regulations and other environmental factors influence cannabis retailers in NL?” the study applies the Comprehensive Cannabis Retail Framework (CCRF), which integrates public health priorities with environmental theory of retail institutional change [6]. The CCRF facilitates a detailed analysis of the perceived barriers to private licensed cannabis retailers and barriers to entry for prospective retailers.

### Retail systems in NL

A key influence on NL’s retail landscape is Canada’s federal framework (the Cannabis Act), introduced in 2018 to restrict youth access, displace illegal markets, and protect public health by enabling regulated adult access [7]. While the Act provides national oversight of production, distribution, and sales, it delegates authority to provinces and territories to design retail systems that reflect local priorities [7]. This decentralized approach has resulted in a diverse mix of retail models across Canada, including government-operated systems, privately managed enterprises, and hybrid structures that integrate elements of both public and private sector involvement [6].

Within this national framework, NL adopted a hybrid four-tiered retail model, with the NL Liquor Corporation (NLC) overseeing regulation, importation, product selection, distribution, price setting, and online sales [8]. NL’s approach aligns with federal objectives but also emphasizes provincial priorities, such as public safety, reducing strain on the criminal justice system, and fostering new business opportunities [9]. However, the success of these goals depends largely on the viability and accessibility of the legal retail market, which is shaped by the province’s regulatory design and implementation.

NL’s model reflects federal priorities but diverges significantly from other provinces. For instance, Manitoba and Alberta operate under more open-market models with fully private retail systems. In contrast, Quebec maintains a government-run monopoly through the Société Québécoise du cannabis (SQDC), emphasizing public health over commercial expansion [10]. Quebec’s model include strict controls on product types, such as banning candy-like edibles, and sets a higher legal age of 21 for purchase, compared to 19 in NL and 18 in Alberta [7,10,11].

NL’s model incorporates private retail participation through a competitive bidding process, where applicants respond to Request for Proposals for specific tiers and locations [12]. Successful applicants must meet rigourous requirements, including security screenings and completing extensive paperwork [12]. While the province’s four-tiered system was designed to balance regulatory control with market accessibility, only two of the four intended tiers are operational [8,13–15]:

**Operational Tiers:**

- **Tier 1 (29 stores):** Standalone cannabis stores with dedicated exterior entrances, restricted access to minors, extensive product selection, and specialized service staff. Retailers earn a 20% commission on sales, may sell cannabis online, and can share non-health-related information.
- **Tier 4 (27 stores):** Convenience-oriented stores selling cannabis alongside other products (e.g., tobacco and alcohol), through a standardized menu. Minors may enter but cannot purchase cannabis. Retailers earn an 8% commission on sales, and all cannabis products must be hidden from view.

**Non-Operational Tiers:**

- **Tier 2:** Intended to feature a dedicated cannabis counter within a non-cannabis retail location. This format has never been implemented.
- **Tier 3:** Envisioned as an enclosed cannabis outlet within a non-cannabis retail space. Only one Tier 3 store, was ever established, and it has since closed.

While NL’s hybrid model shares elements with both private and public systems seen across Canada, its unique tiered structure and centralized wholesale control make it a distinct case. This warrants careful examination, particularly in assessing how well the model supports accessibility, public health, and market sustainability.

### Existing research on the barriers in NL

While NL’s tiered system allows for some flexibility, it also introduces distinct challenges for licensed and prospective retailers, hindering the growth and integration of the cannabis retail market. Structural and operational barriers such as high taxation, a complex and costly licensing process, zoning restrictions, marketing and advertising restrictions, stigma, and limited banking and financing access undermine both existing retailers and potential entrants [16]. Many prospective cannabis retailers, including legacy operators, struggle to secure capital and navigate regulatory hurdles [17,18]. Meanwhile, existing licensed retailers face persistent challenges such as high taxes and restrictions on marketing and financial services [16]. These barriers reduce competitiveness and limit the legal market’s ability to displace unlicensed sales.

Although NL’s cannabis retail market has made progress in reducing unlicensed sales, approximately 16% of cannabis consumption still comes from illegal sources [19]. Price remains a key driver of consumer behavior, with many consumers opting for unlicensed products due to affordability [20,21]. Some jurisdictions have implemented competitive pricing strategies to address this issue. For instance, Quebec has lowered the price of dried cannabis to attract consumers away from unlicensed sources [11,22,23].

However, pricing policies must be carefully calibrated to avoid unintended consequences. Uruguay’s experience offers a cautionary tale: while initially enhancing competitiveness, the government’s decision to match legal market prices with those of the unlicensed market ultimately led to a significant misalignment of demand, resulting in product shortages and rationing [24]. As a consequence, years after legalization, many consumers continue to purchase from illegal sources, undermining the reform’s original objective of eradicating illicit sales [24]. These cases offer important lessons for NL, where price competitiveness and retail accessibility remain key concerns for retailers and policymakers [8,16].

While existing research has primarily focused on consumer transition from the unlicensed to the licensed cannabis market, comparatively little attention has been paid to the structural and operational challenges confronting cannabis retailers [20,21,25,26]. Nevertheless, the long-term viability of the legal cannabis sector depends not only on consumer uptake but also on the ability of licensed retailers to operate sustainably within complex regulatory environments. It also hinges on the capacity of prospective entrants, particularly those transitioning from the unlicensed market, to overcome barriers to entry. This dual focus reflects foundational principles of cannabis market sustainability, which emphasize both operational resilience and inclusive participation as prerequisites for a stable and equitable industry [27]. Without meaningful support for retailers, particularly those from historically marginalized communities, legalization risks perpetuating the very inequities it aims to dismantle, thereby compromising public health and social justice objectives. Yet despite their pivotal role in advancing these goals through retail access and community engagement, the perspectives of licensed and prospective retailers remain largely absent from academic and policy development.

By foregrounding retailer perspectives, this study offers a grounded, stakeholder-informed analysis on how regulatory implementation affects retail viability, alignment with public health goals, and the sustainability of the legal cannabis market. Although situated in NL, the findings may inform broader policy discussions in other jurisdictions seeking to balance public health objectives with the practical realities of cannabis retail market development.

## Methods

### Study design

This qualitative case study employs semi-structured interviews via the Zoom online meeting platform [28] and Ritchie and Spencer’s (2002) five-step framework analysis to examine how government regulations and broader environmental factors shape the experiences of private cannabis retailers in NL. Initially developed for policy research, Ritchie and Spencer’s method supports deductive and inductive reasoning, making it particularly suitable for studies exploring how policy structures are experienced and navigated in real-world contexts [29,30]. Framework analysis offers a structured yet flexible approach that accommodates the complexity of semi-structured interview data [31–33]. In this study, the deductive dimension was informed by the CCRF, which shaped the initial coding structure. Simultaneously, the inductive component allowed for the emergence of context-specific insights from participants’ narratives. Compared to grounded theory, which emphasizes bottom-up theory development [34], or conventional thematic analysis, which may lack a consistent analytic framework [35], framework analysis provided an ideal balance of analytic structure and adaptability. It enabled both the application and refinement of the CCRF while remaining open to unexpected findings.

NL was selected as a single-case study due to its distinctive four-tier cannabis retail system [20], which differs significantly from more centralized or standardized retail models in other provinces [36]. This regulatory divergence offered a unique opportunity to explore how a differentiated market structure interacts with a public health-oriented policy framework. A single-case design allowed for a deep, contextually grounded analysis of these dynamics [37], generating insights that may inform retail policy debates in other jurisdictions.

The study focused on private cannabis retailers, as they serve as critical intermediaries between regulatory institutions and consumers. Licensed retailers were included for their operational experience within the legal framework, while prospective retailers provided insight into market entry challenges. Their perspectives are especially relevant given ongoing concerns about displacing the unlicensed market and ensuring legal market viability. Successful entry and sustainability depend on navigating regulatory barriers and business opportunities, so their experiences are essential to understanding the broader policy landscape [38].

Semi-structured virtual interviews were chosen for their ability to balance consistency across participants with flexibility to explore unanticipated themes [39,40]. This format uses a guiding framework of open-ended questions, allowing researchers to probe deeper into relevant topics while maintaining comparability across interviews [39]. Compared to structured interviews, which follow a fixed set of questions with little room for deviation, semi-structured interviews allow for follow-up questions and adaptation based on participant responses offering greater depth and nuance. In contrast, unstructured interviews are entirely conversational and lack a predefined question set, which can lead to rich, exploratory data but often results in lower consistency and comparability across participants [40]. The semi-structured approach thus provided an optimal balance for this study, enabling focused discussions around core research questions while allowing participants to elaborate on their unique experience navigating the cannabis retail market [39]. Zoom videoconferencing was chosen over phone or in-person methods due to its cost-effectiveness, accessibility, capacity for visual rapport-building, and ability to support real-time, remote engagement with participants across diverse geographic locations [41,42].

### Conceptual framework

**Fig 1:**
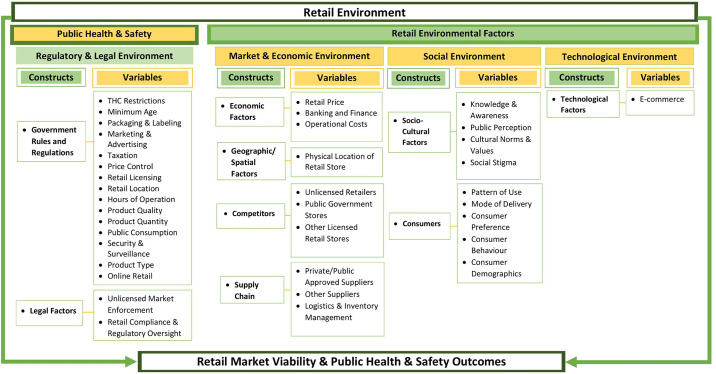
Comprehensive Cannabis Retail framework adapted from Wright-Brown et al. (2024). Wright-Brown, T., Blackwood, M., Cooper, T., Schwartz, E., Newell, W., Bishop, L., Najafizada, M., & Donnan, J., *Examining the Barriers to Licensed Private Cannabis Retailers in Canada: A Quantitative Content Analysis of Canadian News Media Coverage*, *Contemporary Drug Problems* (51 [3]) pp. 202–228. Copyright © 2024 (SAGE Publications). DOI: https://doi.org/10.1177/00914509241271654).

This study applies an adapted version of the Comprehensive Cannabis Retail Framework (CCRF) (Fig 1), a conceptual model introduced by Wright-Brown et al. (2024). Initially developed to assess barriers within the Canadian cannabis retail market, mainly through a quantitative news media content analysis [6], the CCRF offers an integrative approach for understanding how regulatory and environmental conditions shape cannabis retail operations. Its application to a qualitative case study context extends the framework’s utility as a diagnostic tool across multiple data sources and methodological designs.

The CCRF synthesizes two theoretical perspectives: the Canadian Public Health Association’s (CPHA) Public Health Approach to Legalization, Regulation, and Restriction of Access to Cannabis [43], and the Environmental Theory of Retail Institutional Change [44] (Fig 1). The CPHA’s approach prioritizes harm reduction through regulatory interventions, including advertising and marketing restrictions, taxation and pricing controls, limits on THC concentration, packaging and labeling requirements, and strategies to address the unlicensed market [43]. This approach positions legalization as a mechanism to minimize potential health and social harms through comprehensive oversight. Conversely, the environmental theory of retail institutional change focuses on the institutional viability of retail businesses, emphasizing that retailers’ success depends on favorable environmental conditions, which include economic, political, legal, social, demographic, cultural, and technological influences, as well as pressures from competitors, suppliers, customers, labor markets, and financial institutions [44]. Grounded in these dual theoretical perspectives, the study uses the CCRF as an analytical lens and enables a nuanced examination of how cannabis retailers navigate regulatory environments, respond to market pressures, and contribute intentionally or otherwise to public health outcomes.

### Participants and recruitment

Eligible participants were individuals aged 19 or older who either owned or worked in a decision-making role at a licensed retail store or had attempted or intended to enter the legal cannabis market in NL. The age criterion was set at 19, as it is the legal age at which individuals can purchase, consume, or distribute cannabis in NL. A multi-pronged recruitment strategy was employed to ensure a diverse and representative sample, conducted between July 10, 2022, and March 21, 2023.

1. **Direct outreach:** Licensed cannabis retailers were contacted using a publicly available list from Cannabis NL, a division of the NL Liquor Corporation responsible for regulating cannabis sales.
2. **Social media campaigns:** Recruitment posters were shared on Facebook, Twitter, and LinkedIn to reach prospective cannabis retailers.
3. **Snowball sampling:** Participants were encouraged to refer other eligible individuals, thereby expanding the recruitment network.

A total of 27 individuals expressed interest in the study and were sent an informed consent form detailing the study’s purpose, procedures, and participants’ expectations. Of these, 22 met the preliminary eligibility criteria and were invited for an interview. Eighteen participants completed the interview process, nine licensed retailers and nine prospective retailers. Four individuals did not proceed: one was ineligible due to operating outside the study’s jurisdiction, two were unavailable, and one was excluded due to inconsistencies between screening and interview responses, which raised concerns about data reliability. All participants provided informed consent. Some returned signed consent forms via email, while others provided verbal consent at the beginning of the interview. Verbal consents were documented in field notes and witnessed by the secondary interviewer. All consents records were logged in a secure Excel spreadsheet.

### Representativeness of the sample

Of the 18 participants, 17 identified as men and one as a woman, which reflects broader gender trends in Canada’s cannabis retail sector, where men are overrepresented in ownership and leadership roles [45,46]. Most participants were based in urban areas of the province. Among prospective retailers, seven aimed to establish businesses in urban regions, while licensed retailers were evenly distributed between rural and urban locations. This distribution is consistent with national patterns, where cannabis retail stores are more concentrated in urban and socioeconomically diverse areas [47]. All participants completed at least a high school education, with ages ranging from 19 to 49 years.

Most licensed participants operated under Tier 1. However, one Tier 1 participant and the Tier 4 participant operated multiple stores under a corporate banner. When the interviews were conducted, no Tier 2 or Tier 3 stores were operating in NL. Three participants knew their stores were licensed but could not specifically identify their tier (however, we could identify all as tier 1). Additional participants’ characteristics are detailed in Table 1.

**Table 1:** Characteristics of interview participants.

Interview Participants (Pseudonyms) Licensed (n = 9) Prospective (n = 9)	Length of Interview	Characteristics of Participants					
		Gender	Age	Education level	Bus. location	Years in the industry	Business operating years
Pato (licensed – unknown)	41 min.	Male	30-39	High School	Urban/ Rural	4-5 years	2 years
Rex (licensed – unknown)	32 min.	Male	30-39	High School	Rural	3 years	3 years
Chris (licensed – Tier 1)	70 min.	Male	30-39	Graduate	Urban/ Rural	15 years	6 years
Wizzy (licensed – Tier 1)	42 min.	Male	19-29	Undergrad	Rural	5 years	4 years
Jarob (licensed – Tier 1)	55 min.	Male	19-29	Undergrad	Urban	9 years	5 years
Micky (licensed – Tier 1)	60 min.	Male	30-39	Undergrad	Urban	20 years	7 years
Jaki (licensed – Tier 1)	70 min.	Male	30-39	Undergrad	Urban	8 years	4 years
Arsenal (licensed – unknown)	35 min.	Male	30-39	Undergrad	Rural	5 years	5 years
Jennifer (licensed – Tier 4)	44 min.	Female	40-49	College	Urban/ Rural	5 years	5 years
Kenneth (Prospective)	33 min.	Male	30-39	High School	Rural	7-8 years	7-8 years
Mr. Moris (Prospective)	27 min.	Male	19-29	Undergrad	Urban	3-5 years	4 years
Anonymous Wiz (Prospective)	47 min.	Male	19-29	Undergrad	Urban	3.5 years	3 years
Tames (Prospective)	23 min.	Male	30-39	Undergrad	Urban	7 years	5 years
Dave (Prospective)	23 min.	Male	30-39	Undergrad	Urban	4 years	4 years
Constantine (Prospective)	18 min.	Male	30-39	College	Urban	2 years	2 years
Steel (Prospective)	29 min.	Male	30-39	Undergrad	Urban	3 years	1 year
Jewel (Prospective)	35 min.	Male	30-39	Undergrad	Urban	4 years	3 years
Ray (Prospective)	40 min.	Male	19-29	Graduate	Rural	3 years	3 years

### Ethical considerations

Ethical approval was obtained by Memorial University’s Interdisciplinary Committee on Ethics in Human Research (20221951-PH), adhering to the Tri-Council Policy Statement 2 guidelines. Participants received a $25 electronic Amazon gift card upon completing the interview as a token of appreciation.

### Data collection

Semi-structured interviews were conducted via Zoom video conferencing between November 2022 and March 2023, each lasting approximately 30–60 minutes. The lead researcher facilitated the interviews, while a secondary researcher assisted with follow-up questions and field notes. Two interview guides were developed to capture the perspectives of two distinct groups: licensed and prospective retailers. These guides were informed by the CCRF (Fig 1) and were designed collaboratively with the research team. The interview questions explored factors affecting cannabis retailers in NL, experiences with the regulatory process, and barriers or facilitators to compliance with regulatory requirements. All interviews were audio-recorded, transcribed verbatim, and anonymized for confidentiality.

To strengthen the credibility of the findings, saturation was monitored throughout the data collection process. Saturation was considered reached when no new themes or concepts emerged in the final interviews, and existing themes were consistently repeated across participants. This assessment was based on ongoing review of transcripts and field notes by the research team, who met regularly to discuss emerging patterns. The decision to conclude data collection was made collaboratively once it was agreed that additional interviews were unlikely to yield novel insights. This approach aligns with established methods for assessing saturation in qualitative research, which emphasize systematic monitoring and team-based evaluation of theme emergence [48–50].

### Data analysis

Data were analyzed using Ritchie and Spencer’s (2002) five-step framework analysis approach, integrating deductive and inductive strategies to ensure systematic, theory-informed interpretation while remaining responsive to emergent insights. The five steps of the analysis included:

1. **Familiarization:** All transcripts were verified for accuracy and anonymized following automatic transcription in NVivo. The lead researcher engaged deeply with the data, repeatedly listening to audio recordings, reading transcripts, and reviewing field notes to gain an initial understanding of content, patterns, and context.
2. **Identifying a Thematic Framework:** All statements were coded. Deductive codes were derived from the CCRF, while inductive codes were developed from salient issues raised by participants. Initial coding captured significant concepts, phrases, or issues that recurred across the dataset. Initial codes were grouped into broader thematic categories and subthemes based on conceptual similarity and relevance to the CCRF. While the CCRF provided an organizing structure, piloting the framework on a subset of transcripts enabled refinement to enhance clarity, coverage, and contextual fit. This ensured the framework remained flexible enough to incorporate unanticipated yet relevant participant perspectives.
3. **Indexing:** The refined thematic framework was systematically applied to the entire dataset. Transcripts were indexed using an Excel Spreadsheet, enabling consistent categorization across cases. A second researcher coded a sample of the transcripts to assess consistency, with discrepancies resolved through collaborative discussion.
4. **Charting:** Coded data were organized into framework matrices summarizing key excerpts under each theme and subtheme, preserving contextual meaning. This process facilitated systematic comparisons across the dataset, identifying patterns, variations, and relationships among themes.
5. **Mapping and Interpretation of the Data:** The final phase involved systematically reviewing charts and research notes to compare participants’ perceptions and experiences. Rather than just aggregating themes, this stage analyzed patterns to identify relationships and underlying explanations within the broader context of cannabis retail in NL. Findings were presented thematically and descriptively to capture these interconnections, with iterative revisions ensuring coherence and contextual alignment.

### Reflexivity

The interviews were conducted by the first and second authors: a female doctoral student specializing in health outcomes research and a postdoctoral researcher formally trained in qualitative methods. The multidisciplinary research team included professionals from public health, policy, and business backgrounds, none of whom had personal or financial ties to the cannabis industry. There were no prior relationships between the researchers and participants.

The team acknowledged that their professional roles, disciplinary backgrounds, and personal assumptions could influence the research process and interpretation of findings. To mitigate perception bias, a reflexive approach was integrated throughout the study. This included regular team debriefings to surface and critically examine assumptions, the use of open-ended and nonjudgmental interview questions to minimize leading responses, and documentation of researcher reflections during data collection and analysis.

To enhance methodological rigor, the team employed triangulation through field notes and interview transcripts, conducted intercoder reliability checks, and engaged in member checking to validate interpretations. For member checking, participants were invited to review the interview transcripts and provide feedback on the accuracy of interpretations. This process helped ensure that the findings reflected participants’ intended meanings and reduced the risk of misrepresentation. The analysis process was collaborative and iterative, supporting consistency and trustworthiness in the findings.

## Results

Using the CCRF as an analytic lens, this study identified all nine overarching factors and 19 sub-factors influencing the cannabis retail environment in NL. The findings highlight a complex interplay of barriers and facilitators emerging from participants’ accounts, which reflect broader dynamics shaping the province’s cannabis retail landscape. Illustrative participant quotations are included under the respective category, with minor edits for clarity. Pseudonyms are used to protect participants’ anonymity. The results are organized into two main categories that correspond to the study’s core objectives:

- Licensed Retailers’ Reported Barriers and Facilitators to Cannabis Retail Operations in NL
- Prospective Retailers’ Reported Barriers to Entering the Legal Cannabis Market in NL

### Licensed retailers’ reported barriers and facilitators to cannabis retail operations in NL

#### Factor # 1: government rules & regulations.

Seven sub-factors within the government rules and regulations domain of the CCRF were identified as influencing licensed cannabis retailers in NL. Table 2 summarizes perceived barriers and facilitators associated with each sub-factor, as reported during interviews. Numbers in parentheses indicate the number of times participants mentioned the perceived barriers and facilitators. These frequencies reflect the relative prominence of each issue but do not directly measure impact. Though less frequently mentioned, some factors may still significantly influence operations depending on the retailer context and tier.

**Table 2:** Factors relating to government rules and regulations that influence licensed cannabis retailers.

Sub-Factor	Perceived Barrier	Perceived Facilitator
Retail licensing requirements	Rigorous & lengthy process [3]	Mentorship & guidance [14]
	Age-gating policy for tier 1 retailers [1]	Regulatory flexibility for tier 4 retailers [4]
Retail location requirements	Securing a Retail Location [11]	Government Support [1]
Promotion, marketing & advertising restrictions	Advertising & promotion restrictions [10]	Innovative marketing strategies [11]
	Product information sharing restrictions [3]	
	Inconsistent enforcement [1]	
	Community engagement hurdles [1]	
Taxation	High tax rate [12]	Pricing Strategy [10]
Price Control	Fixed pricing [13]	Fixed pricing [3]
Product Type Regulation (Vaping products)	Ban on vaping products [2]	Permitting vaping products [9]
Packaging & labeling restrictions	Packaging challenges [2]	

### Retail licensing requirements

Licensed retailers expressed that obtaining a retail license was challenging, emphasizing the rigorous and lengthy process. However, many said they were able to navigate the process through mentorship and family support. Licensing requirements also vary across retail tiers. For example, a Tier 4 retailer (representing a number of convenience-focused stores) noted that this license enabled their stores to offer a broad range of products, which supported a more integrated business model:

“*We’re a grocery store… people can get their food, their snacks, their alcohol, their cannabis, their cigarettes. We are really a one stop shop, so, our success is definitely based off of that.”* (Jennifer, Licensed Retailer)

Conversely, stricter policies created barriers for retailers with Tier 1 licenses (stand-alone stores), such as age-gating rules that prevented parents with children from entering. The tier 4 retailer also recognized these challenges and advocated for policy alignment between cannabis and liquor stores.

*“In a liquor store, you know, you’re allowed to bring your child in with you. I kind of want to see the same, similar regulations go that way...”* (Jennifer, Licensed Retailer)

### Retail location requirements

Licensed retailers described significant challenges securing suitable retail locations, citing stringent regulatory restrictions. These included postal code limitations, which cap the number of stores allowed within some geographic regions, and proximity rules that prevent stores from operating near schools, parks, or other cannabis retailers. These constraints created a highly competitive and often exclusionary environment, particularly for new entrants. A licensed retailer, summarized these challenges:

*“So, it’s pretty restrictive. They [NL Liquor Corporation] basically put out a collection of postal codes and anybody interested in the public is free to respond with a business proposal.* *They still came with the same restrictions… I keep bringing that up because that does become a hurdle.”* (Chris, Licensed Retailer)

### Promotion, marketing and advertising restrictions

Licensed cannabis retailers operate within a highly regulated environment, particularly regarding promotion, marketing, and advertising, significantly shaping their ability to engage with customers. These regulatory constraints differ across licensing tiers, creating operational disparities within the sector.

Retailers operating under tier-one licenses told us they experience relatively greater flexibility in their marketing strategies than those under tier 4, allowing them to engage in non-health-related discussions about cannabis products. They said this flexibility fosters a more immersive cannabis culture within their stores and enables a customer-focused approach that strengthens brand loyalty and enhances the overall retail experience. However, they mentioned that being unable to provide health-related information presents a challenge, particularly for customers seeking therapeutic or recreational use guidance. One retailer described this constraint:

*“We’re not allowed to give any opinion, or even say, ‘Oh, this cannabis makes you tired.’ You can’t even say that in the store—you could get a ticket from the regulator.”* (Chris, Licensed Retailer)

Licensed tier 1 retailers must rely solely on government-mandated product information, limiting their ability to offer personalized customer support. In contrast, tier four retailers operate under far stricter regulations, significantly restricting their capacity to engage with consumers beyond basic transactional interactions. One retailer illustrated these limitations:

*“We are not allowed to advertise, promote, show anything to the customer..., so that does prohibit us from, you know, really engaging with the customer.”* (Jennifer, Licensed Retailer)

Another major challenge licensed retailers face is the inconsistent enforcement of marketing regulations across the province. A few retailers reported variability in regulatory interpretations by regional inspectors, leading to uneven application of rules, creating compliance challenges. As one retailer described:

*“We’ll have advertising allowed in one area that’s not allowed in the other…because we actually just don’t have one centralized decision maker when it comes to that.*” (Chris, Licensed Retailers)

To navigate these regulatory constraints, some retailers reported they employed creative marketing strategies, sometimes operating in legal gray areas, including promoting their products through social media. Others say they focus on customer retention through incentive-based programs, rewarding high-volume buyers or customers who refer new patrons, despite such practices being restricted under current regulations. As one retailer explained:

*“We’ve always tried as much as we can to keep our customers, especially those that do all their purchases in large quantity. We’ve always given them bonuses.”* (Micky, Licensed Retailer)

Community engagement also plays a role in marketing efforts, with some retailers leveraging philanthropic initiatives to enhance brand visibility and customer loyalty. One retailer recounted their strategy of supporting local organizations:

*“Recommendation helps publicize the business, so we went to a few (youth care homes), gave them clothing, food, and so on. That actually attracted more people… so we kept on selling, and the business kept on expanding.”* (Jarob, Licensed Retailer)

Despite adopting various adaptive strategies, cannabis retailers reported feeling disadvantaged compared to alcohol retailers, who are permitted to sponsor community events and engage in promotional activities. Participants expressed frustration that strict restrictions on cannabis sponsorship limit their ability to build brand awareness and connect with consumers. As one retailer noted:

*“There’s breweries that sponsor concerts and beer gardens and things like that. I think much like alcohol, it’s a social lubricant for many people, and we don’t get the opportunity with provincial legislation.”* (Chris – Licensed Retailer)

### Taxation

Licensed cannabis retailers consistently identified high taxation as a major barrier to competing with the unlicensed market. Participants expressed frustrations that licensed retailers are expected to comply with tax obligations, while unlicensed retailers can offer significantly lower prices. One retailer explained:

*“It’s incredibly difficult to price cannabis to consumers and compete against the black market and the illicit channels out there…All these legacy market people actually do not pay tax and then they can still bring the price per gram down so low that we wouldn’t be able to compete with that. If there was some proportionality there, we would be able to probably follow through with the mandate of a full conversion of legacy market into the legal market. But it’s very difficult in today’s world because of the taxation”* (Chris – Licensed Retailer)

Retailers noted that this pricing disparity discourages consumer transition to the legal market and threatens the long-term viability of legal operations. While some attempted to adopt pricing strategies to remain competitive, the tax burden remained a structural disadvantage. Several participants called for policy reforms, particularly concerning taxation and pricing structures. Such adjustments, they argued, could create a more equitable landscape for legal operators:

“*You need to make sure that you don’t sell too high and also to make sure you don’t sell too low, while at the same time making a profit. If there can be a review on some of these policies that have been set, especially the tax and the price, you know, that would really help.”* (Arsenal – Licensed Retailer)

### Price control

Retailers also raised concerns about the province’s fixed pricing and commission structure, which limited their operational flexibility. In NL, the Newfoundland and Labrador Liquor Corporation (NLC) sets all retail cannabis prices, while retailers earn a sales-based commission tied to volume, initially set at 8% across all tiers. Several participants emphasized that the 8% commission was insufficient to cover basic operating costs, particularly for Tier 4 stores. As one retailer noted:

*“Anybody who runs a retail store in any other industry would know that eight percent is essentially not sustainable.”* (Chris – Licensed Retailer)

Even after lobbying led to an increased 20% commission for Tier 1 retailers, some still found the returns insufficient given the operational demands:

“*Even at 20 percent, they’re not making a profit. The amount of work and demand that I have on my employees almost seems unsustainable*.” (Chris – Licensed Retailer)

In addition to concerns about commission rates, participants discussed how fixed pricing across the province limited their ability to compete, manage inventory, or tailor their offerings. One Tier 4 retailer explained:

*“Having that line pricing across the province does make it very challenging for us to do anything right*. *We’re not really going to be acquiring customers because we have the best price in town. We have the exact same price as the guy down the street.”* “ (Jennifer – Licensed Retailer)

At the same time, the same retailer acknowledged that uniform pricing prevents aggressive price competition and promote fairness, maintaining a level playing field:

*“In Newfoundland, everybody has to be priced the same way. But in other provinces, it’s a race to the bottom.”* (Jennifer – Licensed Retailer)

This highlights a central tension: while standardized pricing and commissions promote fairness and stability, they also restrict competitiveness and profit potential, especially for smaller or lower-volume retailers.

### Product type restrictions (vaping products)

Unlike other cannabis product categories, such as edibles or oils, which were consistently available after legalization, vaping products were initially banned in Newfoundland and Labrador. This regulatory distinction made vaping the only product type mentioned by participants as being restricted at a specific time. Retailers viewed the temporary ban as a significant barrier requiring them to adapt quickly. One retailer described the operational impact:

*“It was potentially devastating to our company at that point, and we had to do a massive pivot and figure out what are the products we’re going to focus on initially.”* (Chris – Licensed Retailer)

The eventual reversal of the ban was seen as a turning point. Several participants reported increased customer demand and improved sales following the introduction of vaping products, which offered an appealing alternative to smoking:

*“It’s on a positive side because we do have customers and clients come in almost every day, different clients, and then our income does increase as well. So, it’s on the positive side.”* (Micky – Licensed Retailer)

### Packaging and labelling restrictions

Health Canada’s stringent packaging and labeling requirements was reported as challenges for licensed retailers, particularly in enabling consumers to assess cannabis quality. Participants expressed frustration over these limitations:

“*It’s very difficult to go into a cannabis shop and make a consumer decision on smell and bag appeal and things like that… because a lot of that has to do with Health Canada compliance or packaging.”* (Chris – Licensed Retailer)

These regulatory constraints hinder retailers’ ability to leverage sensory product evaluation, an important factor in consumer purchasing decisions, further complicating their ability to compete with unregulated markets.

#### Factor # 2: supply chain.

This study identified two supply chain sub-factors: inventory management and logistics, and public suppliers (Table 3).

**Table 3:** Supply chain factors influencing licensed cannabis retailers.

Sub-Factor	Perceived Barrier	Perceived Facilitator
Inventory management & logistics	Inventory management [16]	Multiple suppliers [8]
	Product quality [3]	Positive supplier relationships [5]
Public Suppliers	Centralized warehouse [19]	Centralized warehouse [11]
		Vertical integration model [10]

### Inventory management and logistics

Inventory management was a persistent challenge for licensed cannabis retailers, shaped not only by typical supply and demand dynamics but also by strict regulatory constraints. In NL, all cannabis products must be sourced through the Newfoundland and Labrador Liquor Corporation (NLC), making it illegal to procure inventory outside the regulated system. This centralized sourcing model limits retailer control over product selection and availability. Retailers noted that stock shortages and delivery delays from Cannabis NL negatively affected customer satisfaction. As one participant explained:

*“It’s very unfair for a customer to walk into the stores and can’t find what he or she is looking for”* (Arsenal – Licensed Retailer).

These limitations also impacted retailers’ reputations and perceived reliability

*“Customers feel that I’m not always, you know, meeting up with the demand... So, it has a negative impact.”* (Arsenal – Licensed Retailer)”

Frustrations over inconsistent supply led some participants to consider sourcing from outside the legal system, despite quality concerns and regulatory risks:

*“I get some products that were diluted… the quality wasn’t what I was expecting* (Wizzy – Licensed Retailer)

### Public suppliers

The centralized warehouse system, which was introduced in 2022, further disrupted logistics by severing direct relationships between retailers and producers. Participants reported that this change created additional inefficiencies:

*“Without having that direct relationship that I used to have, I don’t even know who’s buying my product.”* (Chris – Licensed Retailer)

However, vertically integrated businesses, those that both cultivate and sell cannabis, reported greater operational efficiency and consumer engagement:

“*Vertical integration makes my overall company extremely unique… we can ship to our stores directly from our growing facility, which allows us to have a lot of consumer interaction.”* (Chris – Licensed Retailer)

#### Factor # 3: economic factors.

The economic factors represent conditions and trends that may affect the financial stability of cannabis retail operations, including banking and finance (Table 4).

**Table 4:** Economic factors influencing licensed cannabis retailers.

Sub-Factor	Perceived Barrier	Perceived Facilitator
Banking and Finance	Challenges in accessing capital [21]	Financial support from family and friends [13]
	Difficulties obtaining a bank loan [11]	
	Difficulties in opening a bank account [5]	

### Banking and finance

Licensed retailers reported major barriers in accessing financial services, including difficulties with basic transactions like opening a bank account. Many were excluded from traditional banking institutions altogether:

*“Every bank we went to…weren’t even interested in giving us a bank account, let alone putting a mortgage on a building… There are retailers here in Newfoundland right now who still cannot get bank accounts, let alone a business loan.”* (Chris – Licensed Retailer).

To overcome these barriers, some retailers relied on personal loans or family support:

*“My relative gave me money to start up... I would pay him back gradually so that I didn’t involve financial institution.”* (Jaki – Licensed Retailer)

The lack of access to capital also hindered licensed retailers’ operational efficiency, with participants emphasizing the need for government-backed financial assistance:

*“Access to loans would certainly help give other people the opportunity to participate in the industry.”* (Chris – Licensed Retailer)”

#### Factor # 4: Socio-cultural factors.

The analysis revealed two key issues relating to sociocultural factors identified as perceived barriers to licensed retailers: social stigma and cultural norms and values (Table 5). No facilitating factors were identified.

**Table 5:** Socio-cultural factors influencing licensed cannabis retailers.

Sub-factor	Perceived Barrier
Social stigma	consumer skepticism about retailers’ legitimacy [2]
Cultural norms and values	Licensing inequity [1]

### Social stigma

Despite the legalization of cannabis, some retailers continued to face skepticism and doubts about their legitimacy due to lingering social stigma. Community members often perceived them as still engaging in illegal activities, undermining their credibility as licensed businesses. One retailer described these ongoing challenges:

*“There are doubts and fears that I used to operate illegally. They still believe I am operating illegally for some long time.”* (Pato – Licensed Retailer)

### Cultural norms and values

A licensed retailer perceived a lack of diversity and accessibility in the cannabis industry and expressed frustration that legalization had not resulted in equitable participation but instead favored well-resourced entrepreneurs, often from privileged backgrounds, describing this sentiment:

*“There are so many people that feel that they’ve been disenfranchised or excluded from participating in Canadian legalization. It’s not the diversity in Canadian legalization. It’s just a bunch of rich white people.”* (Chris – Licensed Retailer)

Additionally, one participant highlighted the importance of ensuring that gender equity is considered in regulatory frameworks, advocating for fair treatment across gender identities:

*“Another thing that they could have done would have been for them to really consider individuals of different genders. Different genders should have the same rights.”* (Jaki – Licensed Retailer)

#### Factor # 5: consumers.

This study identified two critical factors relating to consumers’ influence: consumer preferences and customer relationships (Table 6).

**Table 6:** Factors relating to consumers’ influence on licensed cannabis retailers.

Sub-Factor	Perceived Barrier	Perceived Facilitator
Consumer Preference	Price Sensitivity [7]	Product quality [11]
	Balancing customer service with operational efficiency [1]	Product diversity [7]
		Timely delivery [5]
Consumer Relationship		Positive customer relationship [24]

### Consumer preferences

Licensed retailers identified several key factors influencing consumer purchasing decisions, including competitive pricing, product quality, product diversity, and efficient service. Price sensitivity was a significant concern for consumers, particularly due to competition from unlicensed markets, as one retailer observed:

*“Some look for alternative where they can get the same goods at the lower prices.”* (Arsenal – Licensed Retailer)

However, the recent decline in legal cannabis prices has become a facilitating factor, making legal products more accessible. As one retailer noted:

*“What I would say as a general statement is the price of cannabis is actually being driven down. Customers really want value. So, the price per gram from when this whole business started to till now has gone down multiple dollars.”* (Jennifer – Licensed Retailer)

High product quality was a crucial differentiator for licensed retailers, helping them attract and retain customers. Retailers emphasized that superior quality set them apart from unlicensed retailers:

*“I think one factor is that they like the quality goods we do sell… because when you offer a good product, you always have customers and people coming to buy.”* (Rex – Licensed Retailer)

Product diversity and frequent menu updates were also highlighted as key drivers of customer loyalty, as they encouraged repeat purchases and sustained consumer interest:

*“They come to our retail stores simply because of the breadth of the menu we have and how frequently it changes.”* (Chris – Licensed Retailer)

In addition to product offerings, efficient service and timely delivery were seen as critical components of consumer satisfaction. The ability to provide reliable and prompt service was regarded as a competitive advantage:

*“I deliver my things at the right and appropriate time… so they are happy because they get their product whenever they want it.”* (Jaki – Licensed Retailer)

While many consumers valued personalized service, one retailer acknowledged operational challenges in balancing individualized attention with business efficiency. They noted that consumer expectations varied, with some preferring quick transactions while others sought more engagement:

*“Some people want to come in and get out. Some people want to talk about cannabis because they love cannabis, and it’s like a kid in a comic shop type of thing, you know? So, figuring out how to appease both is a struggle… Sometimes things get too busy and you can’t do that, and then it’s just kind of a numbers game at that point.”* (Chris – Licensed Retailer)

### Customer relationship

Licensed retailers widely acknowledged that strong customer relationships are crucial to business success. Retailers emphasized the value of friendly and attentive service in fostering consumer loyalty:

*“You’ve got to have friendly retail staff, that’s number one. I hear it immediately if somebody is not treated well... So, you know, you need to focus on your bud tenders [frontline staff], and your staff need to have good customer service. “* (Chris – Licensed Retailer)

A personalized approach to customer engagement was also seen as instrumental in maintaining long-term relationships. Retailers highlighted the role of social interaction in building trust and sustaining consumer interest:

*“The relationship I have with my customer…I think I can make my profit through mutual and social interaction with people.”* (Jaki – Licensed Retailer)

Word-of-mouth referrals and positive consumer feedback emerged as major facilitators of business growth. Long-standing relationships with customers were viewed as an asset in maintaining a loyal consumer base:

*“Some of the advantages I have as a licensed retailer [than some retailers] is that I’ve been in the business for a longer period, and I’ve got testimonies from some of my clients… I’ve always gotten referrals from them.”* (Micky – Licensed Retailer)

#### Factor # 6: competitors.

Participants described the market as intensely competitive, with licensed retailers struggling to differentiate themselves while contending with unlicensed operations that impact their market share and profitability. Two sub-factors were identified: Licensed competitors and unlicensed competitors (Table 7).

**Table 7:** Factors relating to competitors’ influence on licensed cannabis retailers.

Sub-factor	Perceived Barrier	Perceived Facilitator
Licensed competitors	Inability to differentiate themselves in the regulated market [4]	Strategic responses to competition [13]
Unlicensed competitors	Quantity limits [4]	
	Higher price in the legal market [1]	

### Licensed competitors

Due to standardized compliance requirements and uniform product offerings, several licensed retailers described challenges in distinguishing themselves within the regulated market. As one participant explained:

*“We definitely are challenged in finding ways to differentiate ourselves…Because we have tier four in 90 percent of our locations, we don’t have anything that really shows that we’re different from the guy down the street.”* (Jennifer – Licensed Retailers)

One retailer, however, reported greater ability to differentiate, but only because they operated under a unique vertically integrated model not available to other licensed retailers in the province:

*“The menu in my stores sets me apart than the menu at the other retailers”* (Chris – Licensed Retailer)

Retailers also emphasized the importance of pricing in remaining competitive. Despite the NLC’s fixed pricing policy, some participants described informally monitoring competitors’ prices and adjusting where possible within regulatory constraints:

*“The tax and price. It does really bring about a lot of competition. As retailers, we try to find out how much the other person is selling just to, you know, keep in check on all of that.”* (Arsenal – Licensed Retailer)

### Unlicensed retail stores

In addition to competition among licensed retailers, participants consistently viewed unlicensed cannabis sales as a significant threat to their business viability. Multiple retailers pointed to the unlicensed market’s enduring presence and widespread nature. One retailer estimated:

*Maybe 50 percent of all cannabis sales in Canada are still in the legacy market. So, I think what that tells me is it’s not just online sales and delivery that’s doing it… It’s probably that they’re buying more than 30 grams. So, I can only sell 30 grams at a time. But if they’re buying online, they’re probably buying half a pound or quarter pound or full pound and things like that.”* (Chris – Licensed Retailer)

Licensed retailers noted that legal restrictions, such as purchase limits, placed them at a disadvantage, especially when unlicensed retailers operate without such constraints. Some advocated for policy changes to support the legal market:

*“Maybe they can increase the adult possession on that, to maybe 40 grams or 50 grams. It would really go a long way.”* (Jaki – Licensed Retailer)

Others framed the unlicensed market as a threat to the integrity of the legal system and a source of unfair competition for compliant retailers.

*“Competition is really high. There are some people that do so illegally... that’s likely affecting us who have been licensed and who have tried to maintain the rules and regulations. So, in a situation like that, we have been affected in terms of making profits.”* (Jaki – Licensed Retailer)

One retailer went further, characterizing illicit sales as outright theft

“*They are not licensed, and then they’re making money from the business…, that’s an act of theft*.” (Micky -Licensed Retailer)

To remain competitive, some licensed retailers turned to customer retention strategies, including building relationships and extending informal credit to loyal clients:

*“I also give away credit to customers that have been with me for a long time. For new ones, I try to be very friendly with them.”* (Wizzy – Licensed Retailer)

Although contrary to regulations, some also acknowledged adjusting prices and offering promotions to maintain their customer base:

*“We had to slash our prices and sometimes run some free bonuses in order to keep our client.”* (Micky – Licensed Retailer)

#### Factor # 7: legal factors.

A key concern among participants was the impact of law enforcement on the transition to the legal market and the ongoing challenges licensed retailers face (Table 8).

**Table 8:** Legal factors influencing licensed cannabis retailers.

Sub-factor	Perceived Barrier	Perceived Facilitator
Unlicensed market enforcement	Security challenges [2]	Fear of legal consequences [14]
	Limited law enforcement [2]	

Some participants identified the legal risks associated with operating in the unlicensed market, such as the threat of arrest or penalties, as a key motivator for transitioning into the regulated system. As one retailer explained:

*“My main motivation was the security…, the business was kind of patchy, kind of risky.” (Pato -Licensed Retailer)*

However, even within the legal market, safety concerns persisted. Several licensed retailers reported incidents of theft and robbery, prompting investments in enhanced security measures:

*“It was a major problem... I had to tighten up security in our business locations, so that such wouldn’t happen anymore.” (Jarob -Licensed Retailer)*

In addition to these concerns, participants expressed growing frustration with the lack of enforcement against unlicensed operators. They viewed the ongoing presence of illegal sellers as not only unfair competition but also as undermining the integrity of the legal framework. One retailer emphasized the broader implications and called for stronger regulatory interventions:

*“Proper surveillance should be carried out just to make sure that those people understand the demands of the business and to see there is opportunity for them to legalize their business if they really want to venture into it. If they can’t legalize their business, they should just stop it because that’s fraudulent. And at some point, it’s affecting people that have the license and legalize their business. I believe the government can act in one way or another, just to make sure that some people that are unlicensed should be licensed. And I think proper attention should be paid to unlicensed people that are selling illegally.”* (Jaki – Licensed Retailer)

#### Factor # 8: technological factors.

The study identified e-commerce as both a perceived barrier and facilitator to licensed cannabis retailers (Table 9).

**Table 9:** Technological factors influencing licensed cannabis retailers.

Sub-factor	Perceived Barrier	Perceived Facilitator
E-Commerce	Delivery inefficiencies in legal online delivery system [3]	Growth in customer base [1]
	Legacy market dominance in E-commerce [2]	

Licensed cannabis retailers shared varied perspectives on online sales and delivery. For some, these services had little effect on business outcomes, while others viewed them as opportunities to expand their customer base. Despite some optimism, challenges such as delivery inefficiencies and competition from the legacy market were frequently noted. One retailer who launched an online platform described logistical hurdles and limited consumer uptake:

*“We did start an online sales platform with a delivery service, but we can only take the delivery before certain day, and it gets delivered with a bit of a delay. And the uptick on that hasn’t been nearly as great as I thought. The majority of legacy market sales are delivery services and online… mail order marijuana sites, and a lot of them have local delivery drivers. I thought that we would tap into that market, that high volume consumer that’s in the legacy market…I thought maybe we would tap into that. We haven’t seen that yet, but I don’t think my online service is adequate enough.”* (Chris – Licensed Retailer)

In response to these challenges, the retailer proposed integrating real-time delivery tracking, similar to food delivery apps, as a way to enhance the customer experience and shift demand toward the legal market:

*“I think if we had something that was like an app and you see your cannabis coming and it’s right away, then maybe we’ll see something.”* (Chris – Licensed Retailer)

#### Factor # 9: geographic/spatial factors.

The physical location of retail stores was identified as both a perceived barrier and facilitator to licensed cannabis retailers (Table 10).

**Table 10:** Geographical/spatial factors influencing licensed cannabis retailers.

Sub-factor	Perceived Barrier	Perceived Facilitator
Physical location of the retail store	Accessibility challenges in rural areas [1]	Ideal/prime location [3]

### Physical location of the retail store

The physical location of cannabis retail stores emerged as a significant factor influencing business performance, with retailers describing opportunities and limitations tied to geographic context. Those operating in rural areas emphasized the strategic decision to avoid saturated urban markets and to better serve dispersed communities. As one retailer explained, this approach maximized operational efficiency and community impact:

“*The majority of our network is in more rural areas… where we can provide the most bang for our buck essentially, and where it makes the most sense for our stores to service a community.”* (Jennifer – Licensed Retailer)

However, rural settings also presented barriers to access due to NL’s vast geography and low population density. One retailer proposed flexible delivery models to address these gaps and improve equitable access to legal cannabis:

*“In areas that are too rural to operate, I think they need some type of flexibility to deliver to them through a delivery service of some sort…maybe there’s a mobile solution that goes on a weekly route to more rural communities and allows access to safe cannabis on a set schedule.”* (Chris – Licensed Retailer)

Together, these accounts underscore the importance of adaptive retail and distribution strategies, particularly in underserved or remote regions, where standard brick-and-mortar models may not be viable.

### Prospective retailers reported barriers to entering the licensed cannabis retail market

The study identified several factors preventing prospective retailers from entering the licensed cannabis market. These are related to retail licensing requirements, banking and finance, social stigma, cultural norms, and values (Table 11).

**Table 11:** Factors hindering prospective retailers from entering the licensed cannabis market.

Factor	Sub-Factor	Perceived Barrier
Government Rules and Regulations	Retail licensing requirements	Rigorous & lengthy process [10]
		High licensing fees [6]
		Licensing renewal period [6]
		Lack of knowledge about the licensing process [1]
		Perceived favoritism [1]
Economic Factors	Banking and Finance	Access to Capital [8]
		Obtaining a bank loan [1]
Socio-cultural Factors	Social Stigma	Discrimination [2]
	Cultural Norms & Values	Licensing inequity [13]
		Racial & Economic Disparities [12]

#### Factor 1: government rules and regulations.

Retail licensing requirements were the only factor relating to government rules and regulations that participants identified as impacting prospective retailers.

### Retail licensing requirements

Prospective retailers have perceived several barriers to obtaining a cannabis retail license. Several retailers often described the process as time-consuming, complex, and costly. High fees and frequent renewals discouraged some from getting a license, prompting calls for reduced costs and extended license durations. As one prospective retailer shared:

*“I tried once, but the process is quite tedious and long and so ever since then, I have not tried anymore to become a licensed retailer.”* (Anonymous Wiz – Prospective Retailer)

For another, limited access to information and industry networks posed the biggest challenge. Without connections to licensed retailers, understanding the requirements became even more complicated:

*“I didn’t have a good knowledge of how to go about the processes. As someone unlicensed, I always mingled with unlicensed retailers. I had limited interactions with people who had a license, so that made me have limited knowledge of the processes involved.”* (Jewel – Prospective Retailer)

Additionally, some participants expressed frustration with perceived favoritism with the licensing process, where certain individuals seemed to receive preferential treatment from government officials:

*“I’ve been trying for the past one year and some months. I think some of those government guys, they look down on people or they have preferences. Most times you go out there and you can just see... they don’t like you.”* (Ray – Prospective Retailer)

#### Factor 2: economic factors.

The study identified challenges relating to banking and finance as an economic factor that is identified as preventing them from entering the licensed market.

### Banking and finance

Many prospective retailers perceived financial constraints as a key barrier to obtaining a license, highlighting the excessive costs of entering the legal market and the difficulty of securing funding. One prospective retailer shared:

“*I didn’t have… enough funds to get registered... so I’m still working towards that.” (Mr. Moris – Prospective Retailer)*

Some prospective retailers hesitated to seek financial assistance due to concerns about meeting eligibility requirements or fulfilling repayment obligations. As one aspiring retailer noted:

*“I don’t think it would be easy though I haven’t tried getting credit from financial institutions.” (Steel – Prospective Retailer)*

#### Factor 3: sociocultural factors.

Socio-cultural factors associated with social stigma and cultural norms and values were identified as significant influences on prospective retailers’ participation in the legal market.

### Social stigma

Social stigma emerged as a perceived barrier among prospective retailers, particularly concerning sexual orientation. One participant described a friend’s experience in seeking licensing support, suggesting that prejudice based on identity created an unwelcoming environment:

***“****I have a friend who’s also gay, and he didn’t take it funny at all because the moment they knew he was gay, he wasn’t granted assistance at all.”* (Dave – Prospective Retailer)

### Cultural norms and values

Prospective retailers also raised concerns over systemic inequities rooted in race and class. These experiences reflect broader cultural values and institutional practices that shape who is perceived as legitimate or deserving of participation in the legal market. As one participant noted:

*“Because I’m actually Black, and if I walk up to where I’m supposed to get my license and they are all white, I get discriminated for it, and that’s one of my problems.”* (Dave – Prospective Retailer)

To navigate these barriers, some participants considered strategic partnerships with white business owners to improve their chances of approval:

*“I told my friend the other day, we could partner together. He should front the business. I don’t mind signing that agreement with him, though… since I get discriminated due to the fact that I’m Black, I don’t get easier access.”* (Dave – Prospective Retailer)

Economic disparities also played a crucial role in licensing accessibility. Wealthier individuals were perceived as having an advantage, while those with limited resources struggled to meet requirements. One prospective retailer described the financial constraints they faced:

*“Those that have money, they can always get whatever document that is required of them, always work their way through all the hurdles… for us, we have to be very, very careful with the way we spend. And so, it can be discouraging at times.”* (Ray – Prospective Retailer)

Other prospective retailers echoed similar concerns, calling for more inclusive policies to ensure equitable access to licensing:

*“Some persons are being favored while others aren’t given opportunities.” (*Kenneth – Prospective Retailer)

## Discussion

The study applied and iteratively refined the CCRF to analyze the cannabis retail experiences of licensed and prospective retailers in NL. Through framework analysis, the CCRF guided the development of the interview guides and the coding and interpretation of the data. At the same time, the findings informed modifications to the framework, expanding its constructs and variables to better reflect the factors shaping the cannabis retail market in NL. Nine key factors emerged across participants’ accounts, with government rules and regulations, supply chain, economic factors, and socio-cultural factors most frequently referenced as shaping licensed and prospective retailers’ experiences. These factors presented both challenges and opportunities, often reflecting how NL’s distinct regulatory and other environmental context shaped business viability, equity, and market dynamics. Notably, both licensed and prospective retailers reported parallel concerns, especially around the regulatory licensing process, financial access, and experiences of stigma and discrimination. While grounded in NL, these findings may extend beyond NL and may offer valuable lessons for jurisdictions navigating similar cannabis retail market challenges.

### Government rules and regulations

Government rules and regulations, particularly those relating to retail licensing, present significant barriers for licensed retailers to open and operate their retail stores and barriers to entry for prospective retailers. In NL, prospective retailers report considerable challenges, including a prolonged and costly application process, high upfront financial commitments, limited access to capital, and an inequitable license allocation system. Similar barriers have disproportionately impacted entrepreneurs from underrepresented groups in other jurisdictions, such as Ontario, where the high costs of securing a retail license and meeting compliance requirements have been particularly prohibitive [51], a pattern likely to be mirrored in NL. While some licensed retailers have leveraged mentorship and personal networks to navigate these regulatory hurdles, many prospective retailers remain excluded from the legal market due to systemic constraints. Comparable challenges have also emerged in British Columbia, where underfunded and inconsistently implemented initiatives have struggled to integrate legacy operators into the legal market [18,51,52].

The licensing process itself presents further challenges. Lengthy approval timelines, high fees, and short renewal periods deter market entry, a trend also seen in Vancouver, where excessive municipal business license fees have driven some retailers toward the unlicensed market, increasing enforcement costs and heightened public safety concerns [53,54]. Many retailers have advocated for regulatory reforms, including reduced licensing fees and extended renewal periods, to improve accessibility and support small businesses. These advocacy efforts align with broader discussions on reducing entry barriers and fostering a more diverse and inclusive cannabis retail sector [53]. Such reforms could enhance participation among small entrepreneurs while mitigating the persistence of unlicensed sales [55]

Taxation policies further influence cannabis retail prices in NL, where high taxes contribute to legal cannabis being more expensive than unlicensed alternatives. The price disparity has been identified as a major driver of consumer migration to the unlicensed market, consistent with findings in other provinces and international markets [20,56,57]. Research suggests that elevated legal-market prices deter purchases and encourage reliance on the illicit supply chains, particularly in the absence of adequate market stabilization policies [58]. While NL’s price control mechanisms aimed to promote equity, they have been criticized for limiting competition and inadvertently reinforcing consumer dependence on the unlicensed market. A reassessment of pricing strategies, balancing public health objectives with competitive market dynamics, may enhance the sustainability of the legal market while minimizing unintended consequences [2].

### Supply chain challenges

NL’s centralized supply chain model, designed to enhance regulatory oversight and streamline distribution, presents additional challenges for retailers. Delays in product availability and limited direct engagement with suppliers hinder retailers’ ability to meet consumer demand and maintain financial stability. While centralized distribution offers inventory efficiency and cost savings, it also introduces vulnerabilities, such as reliance on a single distribution center and difficulties in responding to market fluctuations [59].

In contrast, decentralized models, such as Saskatchewan’s, where retailers source directly from licensed producers, offer greater autonomy and adaptability [60]. Vertical integration has emerged as a viable approach for overcoming supply chain challenges. In NL, retailers who engaged in both production and retail benefit from direct product delivery, stronger consumer relationships, and improved profit margins. These advantages align with broader supply chain management literature emphasizing customer relationships, profitability, and the ability to tailor offerings to consumer needs [61–63]. However, non-integrated retailers reported difficulty obtaining product variety, securing timely deliveries, and accessing direct delivery options. These concerns highlight the need for flexible, responsive supply chains to maintain a competitive advantage [64,65]. Implementing more flexible supply chain strategies in NL could improve operational efficiency, enhance consumer satisfaction, and foster a more competitive retail ecosystem.

### Economic factors

Economic factors, particularly in banking and finance, pose another significant challenge for cannabis retailers. High startup costs, limited access to capital, and financial institutions’ reluctance to engage with cannabis businesses create substantial barriers to entry. Similar constraints have been observed in the United States and Europe, where federal illegality prevents cannabis businesses from accessing traditional banking services, leading to reliance on cash transactions and increased security risks [66,67]. The lack of financial support for the cannabis industry has been criticized for inadvertently strengthening the unlicensed market [68]. Addressing these challenges through financial policies, such as government-backed loans or partnerships with financial institutions willing to serve the cannabis sector, could facilitate market entry, reduce illicit sales, and promote industry sustainability.

### Socio-cultural factors

Structural and socio-cultural barriers, including racial disparities, economic exclusion, and social stigma, further shape the cannabis retail landscape. Participants in this study described systemic barriers such as racial discrimination and financial exclusion, which constrained their ability to obtain licenses and secure capital. Some individuals were compelled to partner with white business owners in order to navigate these obstacles, reflecting broader disparities in the cannabis industry [18,46,69]. High startup costs, complex licensing procedures, and limited access to capital disproportionately benefit well-connected applicants while further marginalizing Black and Indigenous entrepreneurs [4,69,70].

Social stigma remains another challenge, particularly for minority owned businesses. Persistent prohibitionist narratives contribute to the perception that legal businesses, especially those owned by racial minorities, are suspicious or illegitimate [71]. While some U.S. states, such as New York and Illinois, have introduced social equity programs to address these disparities [72–75], Canada’s initiatives remained insufficient [45,70,76]. These findings highlight the need for Canadian policymakers to explore targeted social equity initiatives to reduce stigma and promote equitable access to the legal cannabis market.

### NL’s tiered cannabis retail model

The study revealed that NL’s unique four-tiered retail model presents regulatory advantages and operational challenges, which differ between tiers 1 and 4. Designed to balance government oversight with accessibility, the system faces inconsistencies in consumer experience, promotional opportunities, and pricing regulations that impact retailer competitiveness. Tier 1 retailers benefit from greater promotional flexibility but struggle with price differentiation due to standardized pricing rules. Meanwhile, Tier 4 retailers can sell a larger variety of non-cannabis products but operate with lower profit margins, raising concerns about long-term sustainability. These findings align with broader critiques suggesting that rigid regulations limit retailers’ ability to build brand awareness, compete effectively, and achieve financial viability [53,77]. While tier 4 retailers argue that standardized pricing promotes equity, the approach has been criticized for weakening competitiveness against the unlicensed market [55,78].

In this context, several Tier 1 retailers described adopting informal and sometimes improvised strategies to navigate regulatory constraints. In the absence of traditional marketing tools, some relied on word-of-mouth promotion, customer loyalty, and community engagement to maintain visibility. Others emphasized staff friendliness and service quality as a way to build trust and retain customers, though these efforts varied widely. A few retailers experimented within regulatory boundaries by tailoring product offerings based on customer preferences or leveraging social media in creative, and occasionally noncompliant, ways. These practices aligns with existing literature, illustrating how some retailers strive to remain viable and responsive within a highly constrained retail environment [79,80].

Consumer experience and accessibility vary significantly across the tiers. Tier 1 retailers reported greater opportunities for consumer engagement, as they can provide detailed product information. In contrast, Tier 4 retailers primarily facilitate transactional interactions, where customer engagements are limited to selecting from a standardized menu, with educational resources restricted to pamphlets or government-approved online references [81]. Accessibility further differentiates these tiers. Tier 1 stores operate as age-restricted environments, reinforcing controlled access but potentially limiting convenience for certain consumer groups. This finding aligns with Health Canada’s report suggesting that provincial and territorial restrictions preventing children from entering cannabis stores may have inadvertently pushed some women, especially those caring for children, to the unlicensed market [55].

Conversely, Tier 4 outlets, embedded within everyday retail environments, offer greater accessibility, particularly for parents and caregivers who may find standalone cannabis stores less convenient. While some Tier 1 stores have introduced drive-thru services to improve accessibility, concerns remain regarding maintaining a safe and discreet purchasing environment [16]. Compared to other provincial models, NL’s tiered system represents a distinct regulatory compromise. Alberta’s open-market system fosters competition and expands consumer choice but raises concerns about the long-term viability of retailers [82]. Meanwhile, Quebec’s government-operated model prioritizes public health objectives, contributing to lower cannabis consumption but often at the expense of market growth and retail profitability [83,84]. NL’s tiered model represents a middle ground, which seeks to balance regulatory oversight with market accessibility. However, its long-term sustainability hinges on addressing operational inefficiencies and refining tier-specific policies to enhance consumer experience and regulatory effectiveness.

### Aligning cannabis and alcohol policies

Participants expressed the desire for greater alignment of alcohol and cannabis policies. While some retailers advocate for harmonizing cannabis regulations with those governing alcohol, the Task Force on Cannabis Legalization and Regulation has recommended stricter measures for cannabis, including advertising restrictions and price controls. This position reflects broader concerns about inconsistencies in alcohol and tobacco regulations, which have historically failed to align with the World Health Organization’s disease risk rankings and the harm potential of different substances [2].

Evidence linking gaps in alcohol policy to significant public health harms supports the Task Force’s cautious stance [85–87]. For instance, alcohol contributes to approximately 17,000 deaths annually in Canada, imposing a greater burden on the healthcare system and society than any other substance [85]. Factors such as low prices, aggressive marketing, and widespread promotion exacerbate consumption, particularly among vulnerable populations like youth [88,89]. Research suggests that exposure to alcohol advertising increases both consumption and initiation, while policy interventions, such as marketing restrictions and higher pricing, effectively reduce alcohol use and related harms [89].

Recognizing these risks, some provinces and advocacy groups in Canada and abroad are strengthening alcohol regulations, as seen in Canada’s updated alcohol consumption guidelines, Ireland’s ban on alcohol ads, and the U.S. Surgeon General’s recent call for cancer warning labels on alcohol [90–94]. While aligning cannabis and alcohol policies may seem logical, it risks replicating the regulatory shortcomings of alcohol. The failures in alcohol regulation highlight the importance of a cautious, harm-reduction approach to cannabis policy. As the Task Force on Cannabis Legalization anticipated, insights from cannabis regulation could eventually inform improvements in alcohol and tobacco policies [2]. This underscores the need for evidence-based, substance-specific policies rather than a uniform regulatory approach.

### CCRF implications for future research

Applying the modified Comprehensive Cannabis Retail Framework (CCRF) in this study demonstrated the value of using an iterative approach to qualitative analysis. The CCRF not only guided the development of the interview guide, coding, and interpretation of the data, but it was also refined in response to empirical findings, expanding several constructs and variables. While the framework encompassed a wide range of variables, not all were prominent in the study’s findings. This reflects the qualitative and context-sensitive nature of the research, where the salience of variables was shaped by participants’ experiences and the specific dynamics of Newfoundland and Labrador’s cannabis retail environment.

The absence of some variables in the data does not diminish their relevance. Instead, it underscores the importance of contextualizing the CCRF to reflect different jurisdictions’ distinct regulatory, economic, and social conditions. Future research could explore how these underrepresented or absent variables manifest in other settings and whether they hold greater explanatory power in alternative cannabis retail markets or policy contexts. This iterative and flexible application reinforces the CCRF’s utility as a guiding and evolving analytical tool.

## Limitations of the study

The study has several limitations. First, the localized focus means that the findings are specific to NL and may not fully capture other jurisdictions’ diverse challenges and opportunities. However, these insights can serve as a foundation for further research, inform local policy decisions, and offer comparative perspectives for other jurisdictions facing similar issues. Second, the study’s participants were primarily men, so the findings may not fully capture the perspectives and experiences of other genders. Future research could benefit from a more diverse sample to better understand how gender influences experiences in the cannabis retail industry. Third, some prospective retailers were hesitant to answer certain questions due to concerns about legal implications. While this presented challenges in obtaining specific information, adherence to ethical guidelines, establishing rapport with participants, and ensuring confidentiality helped encourage candid responses. Future studies may explore alternative data collection approaches, such as ethnography or netnography [95,96], to further address these concerns and enhance participation. Finally, while participants were given detailed instructions for using Zoom, some encountered technical difficulties, causing disruptions during interviews. To mitigate this, participants were given the opportunity to review their transcripts to ensure data accuracy.

## Conclusion

This study examined how regulatory, economic, supply chain, and socio-cultural factors shape the experiences of licensed and prospective cannabis retailers in NL, a province with a distinctive and underexplored retail model. Using Framework Analysis and the CCRF, the study identified perceived persistent and interrelated barriers to market participation and sustainability, including licensing restrictions, taxation, supply chain inefficiencies, financial challenges, and social stigma.

The findings demonstrate how well-intentioned regulations can inadvertently reinforce inequities and hinder market transition if they are not responsive to retailers’ experiences. While NL’s four-tiered model was designed to balance oversight with accessibility, it yielded uneven outcomes across tiers and stakeholder groups. These insights underscore the need for more equitable licensing systems, responsive supply chain strategies, and targeted support for underrepresented retailers. This study also highlights the importance of aligning cannabis policy with public health goals while avoiding the pitfalls seen in alcohol regulation.

By applying and refining the CCRF, this research contributes to cannabis policy scholarship and offers practical guidance for jurisdictions seeking to improve market accessibility, equity, and sustainability. Future research should continue to explore how regulatory environments affect equity-seeking groups and the broader goals of legalization.
